# Was ist Glück? Und was hat die HNO-Heilkunde damit zu tun?

**DOI:** 10.1007/s00106-025-01604-5

**Published:** 2025-04-28

**Authors:** Christian Dobel, Daniel Richter, Orlando Guntinas-Lichius

**Affiliations:** Klinik für Hals‑, Nasen- und Ohrenheilkunde, Universitätsklinikum Jena, Am Klinikum 1, 07747 Jena, Deutschland

**Keywords:** Tinnitus, Cochleaimplantate, Riechstörungen, Fazialisnerverkrankungen, Zwischenmenschliche Beziehungen, Tinnitus, Cochlear implants, Olfaction disorders, Facial nerve diseases, Interpersonal relationships

## Abstract

Glück ist ein Thema, das den meisten Personen in unserer Gesellschaft wichtig ist, obwohl es in der politischen und gesellschaftlichen Zielsetzung wenig Bedeutung bekommt. Glück ist jedoch ein bedeutendes Thema in diversen philosophischen Schulen und geht in der westlichen Philosophie auf den Aristotelismus und die stoische Philosophie zurück. Aktuelle Längsschnittstudien legen sehr klar nahe, dass Glück von zwischenmenschlichen Beziehungen abhängt. Was hat nun die HNO-Heilkunde damit zu tun? Aus unserer Sicht stehen im Zentrum der HNO-Heilkunde diverse Basisfähigkeiten, die Menschen erst ermöglichen, soziale Fitness zu erlangen, d. h. kurz- und längerfristige Beziehungen zu knüpfen. Beispiele dafür sind das Hören, der Geruchssinn und die Stimme, aber auch Gesichtsbewegungen. Durch die multisensorische Verarbeitung diverser Wahrnehmungs- und Ausdruckskanäle entsteht im Gehirn ein „social brain network“ als neuronales Korrelat sozialer Fähigkeiten und Fitness. Wir propagieren, dass die HNO-Heilkunde für das Verständnis von diesen Fähigkeiten und Glück eine zentrale Rolle einnehmen sollte.

## Abstract (en)

Happiness is important to most people in our society, although it is given little importance in political and social objectives. However, happiness is an important topic in various philosophical schools and in Western philosophy, happiness goes back to Aristotelianism and Stoic philosophy. Recent longitudinal studies clearly suggest that happiness depends on interpersonal relationships. So what does otorhinolaryngology have to do with this? In our view, various basic skills that enable people to achieve social fitness, i.e., to establish short- and long-term relationships, are at the center of ENT medicine. Examples of this are hearing, olfaction, the voice, but also facial movements. The multisensory processing of various perception and expression channels creates a “social brain network” as a neuronal correlate of social skills and fitness. Consequently, otorhinolaryngology should play a central role in understanding these abilities in the context of happiness.

## Tinnitus und die Suche nach Glück

Das Thema „Glück“ entwickelte sich bei uns durch die Gespräche mit unseren Patientinnen und Patienten in unserer Tagesklinik für chronischen Tinnitus [51]. Sehr häufig wurde auch von uns in bei dieser Störung empfohlen, man solle sich ablenken von dem Ohrgeräusch. Problematisch ist, dass die Patientinnen und Patienten dann in weitere energieverschlingende Anstrengungen geraten, die nur neue Belastung verursachen. Wir formulieren das deshalb mittlerweile anders und raten unseren Patientinnen und Patienten sie mögen tun, was sie tun wollen. Daraus ergibt sich dann häufig ein Gespräch zu den Zielen und Wünschen im Leben und wie man die Person wird, die man sein möchte. Früher oder später fällt das Wort „Glück“. Vor dem Hintergrund wollten wir wissenschaftlich aufarbeiten, wie wir unsere Patientinnen und Patienten besser unterstützen können. Die Literatur dazu ist beträchtlich und wurde auch in aktuellen populärwissenschaftlichen Büchern von Medizinern und Wissenschaftlern auch vor dem Hintergrund unterschiedlicher Kulturen aufgearbeitet [37, 74, 101]. Erkenntnisse daraus flossen mittlerweile in unsere Therapieansätze ein, aber wir erkannten schnell, dass die Thematik allgemein für die HNO‑Heilkunde relevant ist.

Qualität und Quantität zwischenmenschlicher Beziehungen sind wesentlich für das Erleben von Glück

Zum Beginn des Artikels geben wir eine kurze philosophische Einordnung in das Thema Glück. Im Anschluss werden wir das Thema vor dem Hintergrund von Längsschnittstudien beleuchten. Vorausschauend sei gesagt, dass die Qualität und Quantität zwischenmenschlicher Beziehungen ein wesentlicher Aspekt für das Erleben von Glück ist. Dementsprechend stellen moderne neurowissenschaftliche Theorien ein „social brain network“ vor, das durch multisensorische Verarbeitung charakterisiert ist und das neuronale Korrelat für die Erlangung sozialer Fertigkeiten und sozialer Fitness darstellt. An unterschiedlichen Beispielen aus der HNO-Heilkunde erläutern wir, dass sie eine zentrale Rolle beim Gewinn und Erhalt dieser Fähigkeiten einnimmt.

## Philosophische Einordnung

Glück ist ein Thema mit klassischen Wurzeln. Die systematische Suche nach einem adäquaten Konzept des menschlichen Glücks und dessen konkrete lebenspraktische Verwirklichung im Dasein des Einzelnen ist eine zentrale Größe der klassischen Philosophie (in diesem Artikel fokussieren wir auf die klassische Philosophie, aber es gibt natürlich auch Ansätze aus der Moderne [95]). Die überlieferten Denkschulen verband dabei das Ziel der Ausformulierung eines als praktische Lebenskunst verstandenen Glücksbegriffs [44]. Aus der vorliegenden Perspektive ist zu klären, ob sich in jenen Überlegungen zur Lebenskunst bereits eine Art Entsprechung für die moderne Idee des „social brain network“ findet.

Grundlegend muss festgehalten werden, dass der Glücksbegriff in der klassischen Periode eine stark intellektualisierte, nach innen gerichtete Tendenz aufwies. Mit Ausnahme der Denkschule der Kyrenaiker, welche der *Hedonia *als gegebenem Lustempfinden einen höheren Stellenwert beimaß [26], zeigte sich in den übrigen klassischen Positionen deutlich der Primat eines als *Eudaimonia *verstandenen Glücks. Letztere wurde übergreifend als ein sich vorwiegend oder gar allein im rein vergeistigten Streben zeigender Erfüllungszustand aufgefasst [46]. Der Vorzug des Erfüllungsglücks gegenüber der hedonistischen Position des Empfindungsglücks schien dabei zunächst mit dem Versuch verbunden, den Zustand des Glücks und mithin das erkennende Subjekt als seinen Adressaten gegenüber allen äußeren Einflüssen und Widrigkeiten zu immunisieren. So war für Platon Glück ein Glück des Gerechten und gerecht sei der, der „gut lebt“. Der Philosoph erhalte dabei seine Gerechtigkeit rein aus der Betrachtung der Ideen als etwas Wohlgeordnetem und Gleichbleibendem und dem Imitieren der sich in ihnen zeigenden Ordnung [106]. Jenes allein an der Erkenntnis orientierte Leben und der darin konturierte Geistes- oder Seelenzustand schien prima facie weit entfernt von einer Position, welche sozialen Größen und ihrer möglichen Rolle für die nach Glück strebende Lebenskunst in höherem Maße Rechnung trägt. Doch bereits im Denken des Schülers Aristoteles ist eindeutig eine Anerkennung dessen erkennbar, dass der Mensch als ein nach *Eudaimonia* strebendes Individuum nicht aus seinem sozialen Bezugsrahmen gelöst betrachtet werden kann. Im aristotelischen Denken ist die *Eudaimonia* als abschließendes Strebensziel, als hinreichend für ein gutes Leben und für sich genommen als nicht verbesserungsfähig ausgewiesen [45].

Der sich widerspiegelnde Gedanke der Autarkie und vollständigen Selbstgenügsamkeit wurde in der näheren inhaltlichen Bestimmung mit einer Güterlehre verbunden, welche die möglichen Güter auf ihre glückskonstituierende Rolle hin abwog. Konstituierend für die *Eudaimonia* sind für Aristoteles dabei nur Güter, die um ihrer selbst willen und nicht um anderer Güter willen wählenswert erscheinen. Die darin enthaltene Aufwertung intrinsisch wählenswerter Güter aufgrund ihrer Relevanz für die *Eudaimonia* zeigt sich in seinen Werken an verschiedenen Stellen. Interessant dabei ist, dass die sozialen Güter, allen voran die Freundschaft, stets in die Gruppe der Glückskonstituenzien fallen [87]. Noch deutlicher als in seiner *Rhetorik* weist Aristoteles die tragende Rolle sozialer Güter in der *Nikomachischen Ethik* aus. So schreibt er: „… denn mit dem Glück eines Menschen ist es schlecht bestellt, wenn er ein ganz abstoßendes Äußeres oder eine niedrige Herkunft hat oder ganz allein im Leben steht und kinderlos ist. Noch weniger kann man vom Glück sprechen, wenn jemand ganz schlechte Kinder oder Freunde besitzt oder gute durch den Tod verloren hat …“ ([4] NE 1099b 2–8). Nimmt man dies wörtlich, so scheint ein gemäß der *Eudaimonia* gelingendes Leben für Aristoteles auch zu wesentlichen Teilen ein gelingendes soziales Leben zu sein, welches eindeutig graduell verstandene, qualitative Aspekte aufweist. Dabei dürfte es wohl unstrittig sein, dass ein Gelingen der Kindeserziehung oder die Pflege guter Freundschaften untrennbar mit der Erlangung eines hinreichenden Maßes an sozialer Fitness verbunden ist. Vieles spricht dafür, dass kein Geringerer als Aristoteles im Kontext einer modernen neurowissenschaftlichen Perspektive ein durch adäquaten Input etabliertes „social brain network“ entweder als Teil oder zumindest als notwendige Bedingung dessen erachtet hätte, was als *Eudaimonia *bereits alles in sich Erstrebenswerte enthält.

Der Umfang der aktuellen Forschung zum Thema Glück ist nicht gering und wird in der World Database of Happiness gesammelt [99]. Die verwendeten Maße (meist einfache Fragen) unterscheiden sich stark, können aber auch grob in 2 Gruppen klassifiziert werden: ein affektives Maß, in dem erfragt wird, wie gut man sich zu einem Zeitpunkt oder die meiste Zeit fühlt (grob: hedonia), und ein eher kognitives Maß, wie sehr man meint, vom Leben zu bekommen, was man will (grob: eudaimonia) [98].

## Erkenntnisse aus Medizin, Psychologie und Menschheitsgeschichte

Zum Thema Glück gibt es Erkenntnisse aus Längsschnittstudien, die aus der medizinischen Forschung, der Psychologie und der Menschheitsgeschichte stammen. Mehrere Längsschnittstudien aus unterschiedlichen Teilen der Welt analysierten das Thema eines guten und erfolgreichen Lebens. Die Daten stammen aus Großbritannien [86], Neuseeland [79], Niederlande [78] und den USA [9, 103]. Vielleicht am eindrücklichsten wurde das anhand der Harvard Study of Adult Development erarbeitet, die seit über 80 Jahren weitergeführt wird [31, 52]. Die Ergebnisse wurden kürzlich in einem populärwissenschaftlichen Buch aufgearbeitet [101].

Übereinstimmend kommen diese Studien zu dem Schluss, dass Menschen, die enge Beziehungen zu ihrer Familie, ihren Freunden und der Gemeinschaft haben, insgesamt glücklicher sind, aber sich auch einer besseren Gesundheit erfreuen. Personen, die am zufriedensten in ihren Beziehungen in ihren 50er-Jahren waren, waren im Alter von 80 die gesündesten, sowohl physisch als auch psychisch [96]. Personen im Alter von 80 Jahren hatten ein stabiles Glücksempfinden, obwohl sie unter stärkeren Schmerzen litten, aber nur dann, wenn sie in glücklichen Partnerschaften lebten. Das steht im Gegensatz zu Personen in unglücklichen Partnerschaften, die bei höheren Schmerzen auch unter schlechterer Stimmung litten [102]. Soziale Unterstützung und befriedigende Beziehungen wirken sich ebenfalls positiv auf depressive Symptomatik aus, wie in den genannten Längsschnittstudien aus den Niederlanden, Neuseeland und Chicago/USA berichtet wurde [10, 78, 79].

Menschen, die enge Beziehungen zu Familie, Freunden und der Gemeinschaft haben, sind glücklicher und gesünder

In einer viel beachteten Studie in Science [47] berichten die Autoren von einem engen Zusammenhang zwischen Mortalität und sozialer Integration. Spannenderweise wurde das bei sehr unterschiedlichen Populationen festgestellt, sowohl bei weißen und afroamerikanischen Personen aus Evans County/Georgia, aus Tecumseh/Michigan, Alameda County/Kalifornien, Gothenburg/Nebraska oder Ost-Finnland. Obwohl sich die Entwicklungs- und Lebensbedingungen in diesen Regionen sehr stark für die untersuchten Populationen unterscheiden, war die soziale Integration ein maßgeblicher Faktor für alle und auch über alle Altersgruppen hinweg. Dieser enge Zusammenhang wurde auch in anderen Ländern, z. T. in Längsschnittstudien und klinischen Populationen, gefunden [34, 43, 60, 82]. Zu diesen zwischenmenschlichen Beziehungen zählen nicht nur Familie und Partnerschaften, sondern auch gute Kontakte innerhalb des Berufslebens, die die Bewältigung der Arbeit deutlich erleichtern [75]. Auch sehr kurze, „unwichtige“, aber positive Kontakte, z. B. mit dem/der Barista beim Erwerb eines Kaffees, führen zu positivem Affekt und einer Erhöhung der Stimmung [89]. Wenn sich diese kurzfristigen Kontakte mit Fremden wiederholen, können sich daraus lockere Freundschaften entwickeln [36]. Die Wichtigkeit für das Befinden von lockeren, kurzfristigen und schwachen („weak ties“) Beziehungen zu eigentlich fremden Personen sollte nicht unterschätzt werden und konnten experimentell nachgewiesen werden [29, 35].

Vor diesem Hintergrund ist es nicht verwunderlich, dass sozialer Ausschluss ähnliche Hirnareale aktiviert, wie das bei physischem Schmerz beobachtet wird [77]. Experimentell wurde dieser Effekt während neurophysiologischer Messungen, vorranging funktionelle Magnetresonanztomographie (MRT), untersucht während die Teilnehmer „Cyberball“ spielten, ein Computerspiel, indem durch experimentelle Manipulation die Versuchspersonen entweder eingebunden oder ausgeschlossen werden [38].

Betrachtet man die Geschichte des Homo sapiens, die vor 6–7 Mio. Jahren begonnen hat, dann wird sehr schnell klar, dass unsere Vorfahren nie allein waren, sondern dass die hohe Anpassungsfähigkeit durch das soziale Gruppenverhalten entstanden ist [59]. Eine der Grundüberzeugungen in den kognitiven Neurowissenschaften ist, dass unser Gehirn genetisch vorbereitet ist, bestimmte Inhalte zu erlernen, wie z. B. Objekterkennung und Numerosität, aber auch sozial relevante Funktionen wie Sprache, Gesichterwahrnehmung, Erkennen von emotionalen Zuständen usw. Dies mündet in einem „social brain“ [3] bzw. „social brain network“ [53], wodurch sehr schnell ablaufende Prozesse wie das Erkennen von Gesichtern, aber auch langsamere Prozesse wie die Evaluation emotionaler Prozesse anderer Personen geleistet werden. Diese Netzwerke stellen eine Verbindung unterschiedlicher Hirnregionen dar, die bei sozialer Wahrnehmung und entsprechenden kognitiven Aufgaben aktiviert werden. Derartig komplexe Funktionen können nicht von einzelnen isolierten Regionen erbracht werden, sondern durch Netzwerke, die multisensorisch organisiert sind, wodurch generell die Funktionsweise des Neokortex gekennzeichnet ist [33]. Vor diesem Hintergrund entstand das Konzept der „sozialen Fitness“, d. h. die Struktur des sozialen Lebens einer Person und die Interaktionen eines Individuums innerhalb von Beziehungen und Gruppen [11]. Insbesondere bei Gruppen, die starkem Stress und erheblicher physischer Belastung ausgesetzt sind, wie z. B. im Militär, ist bewusst geworden, wie sehr Gruppenkohäsion die Resilienz erhöht [14]. Freundschaften und positive zwischenmenschliche Beziehungen sind ein protektiver Faktor für extrem stressbelastende Situationen wie Kriegshandlungen und schützen vor posttraumatischer Belastungsstörung, wie an Veteranen aus dem ersten und zweiten Weltkrieg gezeigt wurde [76].

Das menschliche Gehirn ist vorbereitet auf soziale Interaktionen, allerdings nicht auf Einsamkeit

Das menschliche Gehirn ist also von Geburt an vorbereitet auf soziale Interaktionen, allerdings nicht auf Einsamkeit. Das kann gut die dramatischen Konsequenzen von Einsamkeit erklären. Eine metaanalytische Übersichtsarbeit zeigte, dass Einsamkeit die Wahrscheinlichkeit der Mortalität um etwa 26 % erhöht, unabhängig von Geschlecht, objektiv oder subjektiv empfundener Isolation oder weltweit erhobener Region [42]. Generell geht Einsamkeit mit schlechterer Gesundheit in diversen Bereichen einher [64, 72].

Was hat nun die HNO Heilkunde damit zu tun?

## Rolle der HNO-Heilkunde

### Hören

Immanuel Kant wird der Satz zugeschrieben „Nicht sehen trennt von den Dingen, aber nicht hören trennt von den Menschen“, und dieser ist sicherlich jeder HNO-Ärztin und jedem HNO-Arzt geläufig. Er erläutert am besten, worauf wir in dem vorliegenden Artikel abzielen. Obwohl die Bedeutung des Satzes jeder vorgebildeten Person aus der HNO-Heilkunde bekannt sein dürfte, ist Altersschwerhörigkeit ein unterschätztes Problem mit weitreichenden Folgen. Das betrifft nicht nur Deutschland. Altersschwerhörigkeit ist ein globales Gesundheitsproblem, das weitaus häufiger auftritt, als es diagnostiziert und behandelt wird. Etwa ein Drittel der Menschen über 65 Jahre leidet weltweit an Altersschwerhörigkeit, und bei den über 80-Jährigen steigt die Zahl auf 43 % [80, 81]. In Deutschland zeigen Studien sogar noch höhere Prävalenzen: In der Gutenberg-Studie wiesen 34,5 % der Probanden eine beidseitige Schwerhörigkeit und 47,7 % eine Hörgeräteindikation auf, wobei nur 7,7 % tatsächlich ein Hörgerät trugen [24]. Die Folgen von Altersschwerhörigkeit sind vielfältig und schwerwiegend: Sie kann zu sozialer Isolation, Depressionen, Demenz, Stürzen und einer verminderten Lebensqualität führen, d. h. Erkrankungen mit hoher individueller und volkswirtschaftlicher Krankheitslast [5, 18, 66, 91].

Die gute Nachricht aus Sicht der HNO-Heilkunde ist, dass Altersschwerhörigkeit ein modifizierbarer Risikofaktor ist. Durch gezielte Maßnahmen können die negativen Folgen beeinflusst werden. Die Behandlung der Altersschwerhörigkeit hat im Vergleich zu anderen Lebensstilfaktoren das höchste Potenzial, die Demenzprävalenz weltweit und in Deutschland zu senken [5, 65].

Hörgeräte können bei vulnerablen Gruppen kognitiven Abbau vermeiden und das Demenzrisiko verringern

So zeigen aktuelle Studien, dass Hörgeräte bei besonders vulnerablen Gruppen den kognitiven Abbau vermeiden und das Demenzrisiko kompensieren können [65]. Darüber hinaus führen Screening-Programme für Altersschwerhörigkeit zu einer deutlichen Erhöhung der Hörgeräteversorgung und senken die Folgekosten [16]. Die Weltgesundheitsorganisation (WHO) empfiehlt daher ein reguläres Hörscreening für alle älteren Menschen [81]. Als vulnerable Gruppe seien hier nicht nur ältere Menschen genannt, sondern z. B. auch Teilnehmer der „Special Olympics“, bei denen ein erhöhtes Risiko für Hörbeeinträchtigung festgestellt werden konnte [41].

Neben Hörgeräten stellen auch Cochleaimplantate eine vielversprechende Möglichkeit dar, um Schwerhörigkeit bei Kindern und Erwachsenen auszugleichen. Aktuell sind dies die erfolgreichsten Neuroprothesen zur Verbesserung der Hörfähigkeit [55, 104, 107]. Cochleaimplantate führen zu besserem Sprachverstehen [83, 84], wobei multifaktorielle Modelle für die Prädiktion der Verbesserung am besten geeignet sind [63]. Die Versorgung mit Cochleaimplantaten führt zu reduzierten kognitiven Einbußen, wie in einer metaanalytischen Studie gezeigt wurde [1]. Während verständlicherweise das Sprachverständnis in den vergangenen Jahren im Zentrum der Forschung und Versorgung stand, gewinnt in den letzten Jahren die Verarbeitung von nonverbalen und emotional-sozial relevanten Reizen, die über die Stimme von Sprecherinnen und Sprechern vermittelt wird, immer mehr an Bedeutung [32, 73, 90, 92]. Die gute Nachricht ist auch hier, dass es effektive Trainingsmöglichkeiten für emotional-soziale Reize nach der Versorgung gibt [100]. Auch in diesen Trainingssituationen scheinen crossmodale Prozesse durch auditive und visuelle Stimulation gleichzeitig zum Erfolg zu führen [2].

Hören ist eine der Grundvoraussetzungen für zwischenmenschliche Kommunikation, den Erwerb von sprachlichen Funktionen und damit für menschliche Beziehungen. Innerhalb der medizinischen Fachdisziplinen hat die HNO-Heilkunde ein Alleinstellungsmerkmal in der Versorgung bei Hörverlust über die gesamte Lebensspanne. Es soll an der Stelle nicht unerwähnt bleiben, das auch Gebärdensprachen eine Option bei Taubheit sind, insbesondere von Geburt an, wenn die Prägung auf eine Muttersprache noch nicht erfolgt ist. Gebärdensprachen teilen viele strukturelle Gemeinsamkeiten mit gesprochenen Sprachen, aber unterscheiden sich in anderen Aspekten [67, 88], u. a. durch die räumlichen Parameter der Sprache [20, 28]. Da beim Verständnis von gesprochenen und gebärdeten Sprachen überlappende Bereiche des Sprachnetzwerks aktiviert werden [28], darunter am eindrucksvollsten das Broca- und Wernicke-Areal [68], stellen sie ein schönes Beispiel für „Hören mit den Augen“ dar [12, 23]. Wie bei gesprochenen Sprachen ist crossmodale Verarbeitung ein zentraler Aspekt zur Wahrnehmung sozial-emotionaler Reize [22].

### Riechen

Lange Zeit wurde der Geruchssinn vernachlässigt und seine Bedeutung unterschätzt. Dabei spielt er eine zentrale Rolle in menschlichem Leben für die gesamte Lebensspanne, indem er uns auf vielfältigen Ebenen unterstützt, wie z. B. bei der Vermeidung von Umweltgefahren, da uns Gerüche vor giftigen Substanzen, Rauch, verdorbenem Essen und anderen Gefahren warnen. Der Geruchssinn unterstützt uns, Lebensmittel zu finden, zu beurteilen und zu genießen und trägt somit zu einer gesunden Ernährung bei [7]. Schließlich und für das aktuelle Thema relevant, sind olfaktorische Reize wichtige Faktoren für das körperliche Wohlbefinden, die Stimmung und die zwischenmenschliche Kommunikation [69].

Kongenitale Anosmien betreffen auch zwischenmenschliche Beziehungen

Sie spielen eine wichtige Rolle in der nonverbalen Kommunikation und schaffen emotionale Verbindungen. Gerüche helfen, die Mutter-Kind-Bindung zu etablieren [15], und spielen eine zentrale Rolle bei der Partnerwahl [39, 40, 71]. Das betrifft die Initiierung, Aufrechterhaltung und den Verlust von romantischen Beziehungen. Kongenitale Anosmien führen wiederum zu weniger festen romantischen Beziehungen [70].

Riechfunktionsstörungen beeinträchtigen diese 3 Säulen und führen zu erheblichen Einschränkungen in der Lebensqualität [48]. In einem systematischen Review wurde eine enge Beziehung zwischen Depression und Riechen etabliert: Patientinnen und Patienten, die unter Depressionen leiden, zeigen auffällige Riechleistungen, und umgekehrt leiden Patientinnen und Patienten mit Anosmien häufiger unter depressiven Symptomen [58] und unter einem beeinträchtigten Sozialverhalten [7]. Riechstörungen führen allerdings auch zu einem verringerten Genuss von Essen und Trinken. Die Freude am Essen und Trinken kann stark beeinträchtigt sein, da Aromen nicht mehr wahrgenommen werden können [6]. Dies wiederum beeinträchtigt die Lebensqualität erheblich [7].

Riechen und Riechstörungen sind ein zentrales Thema in der HNO-Heilkunde. Die Konsequenzen von Riechstörungen betreffen vorrangig auch zwischenmenschliche Beziehungen, sowohl im frühkindlichen Alter als auch bei Erwachsenen.

### Mimik

Unser Gesicht spricht Bände über uns, bevor wir überhaupt ein Wort sagen. Das Gesicht spielt eine Schlüsselrolle bei der Affektdarstellung, d. h. der äußeren Darstellung der eigenen Emotionen als wichtigen Bestandteilen der verbalen und nonverbalen Kommunikation. Glück („happiness“) gehört nach Ekman zu den 6 universellen Basisemotionen [27]. Ein glücklicher Gesichtsausdruck ist auch von grundlegender Bedeutung für das eigene Wohlbefinden und die Fähigkeit, sich in ein soziales Netzwerk zu integrieren [8]. Wichtig ist hierbei der psychologische Begriff der emotionalen Ansteckung („emotional contagion“). Dies beschreibt die Fähigkeit, einerseits eine bestimmte Stimmung („ich bin glücklich, zufrieden“) zu vermitteln, eine bestimmte Stimmung zu kommunizieren und empathisch auf andere Menschen einzugehen [30].

Daher ist es naheliegend, dass eine gestörte mimische Ausdrucksfähigkeit, wie dies bei einer Fazialisparese vorliegt, zu bedeutsamen sozialen und emotionalen Störungen führt [21, 56]. Die erheblich geminderte Lebensqualität bei Patienten mit Fazialisparese geht weniger zurück auf die rein motorische Funktionsstörung als auf die Stigmatisierung und eben die eingeschränkte emotionale Ausdrucksfähigkeit [13, 97]. Wenn man Patienten mit chronischer Fazialisparese ohne oder mit Synkinesien fragt, was sie am meisten stört, so wird insbesondere von Frauen als Erstes der Verlust, lachen zu können, genannt. Zeigt man Laien Bilder von Patienten mit Fazialisparese, so werden Gesichter mit einem Lachen weniger häufig als positiver emotionaler Ausdruck wahrgenommen als bei Bildern von gesunden Menschen [49, 50]. Patienten mit Fazialisparese werden als weniger attraktiv wahrgenommen. Lachen steigert anders als bei Gesunden die Attraktivität nicht [49]. Damit einher geht auch, dass Patienten mit Fazialisparese weniger vertrauensvoll eingeschätzt werden [85]. Unsere Einschätzung ist also ganz anders bei einem Patienten mit Fazialisparese als z. B. bei Menschen, die Botulinumtoxin-Injektionen gegen Falten erhalten haben. Auch Botulinumtoxin führt letztendlich zu einer geringeren Gesichtsbewegung. Hier wird die Maßnahme aber als attraktiv eingeschätzt [19]. Bei schlaffer Fazialisparese kann eine chirurgische Fazialisreanimation die Attraktivität, insbesondere beim Lachen, steigern [17]. Interessanterweise können Laien häufig nicht die Seite der Lähmung benennen. Es scheint also so zu sein, dass wir unsere Einschätzung des emotionalen Ausdrucks unseres Gegenübers mehr holistisch einschätzen, als bewusst einzelne Gesichtsregionen zu interpretieren [94].

Der verminderte emotionale Ausdruck hat erhebliche Auswirkungen auf soziale Interaktionen

Der verminderte emotionale Ausdruck hat auch Auswirkungen auf die Emotionserkennung und zerebrale Verarbeitung von Emotionen [54]. Wichtig ist hier die Theorie der „embodied cognition“. Diese besagt, dass ein wichtiger Mechanismus zur Wahrnehmung von Gesichtsemotionen deren sensomotorische Simulation im eigenen Gehirn ist. Dies bedarf einer mentalen Nachbildung des wahrgenommenen Gesichtsausdrucks [56]. Wenn also der Gesichtsausdruck gestört ist, ich also nicht glücklich aussehen kann, verändert dies auch die mentale Nachbildbarkeit eines Glücksausdrucks meines Gegenübers, was letztlich die Fähigkeit zu Simulation und damit zur Erkennung dieser Gesichtsemotion einschränkt [105]. Ob dies generell gilt, kann noch nicht abschließend beurteilt werden. Es gibt auch Untersuchungen, die keinen Einfluss einer Fazialisparese auf die Fähigkeit, Emotionen anderen erkennen zu können, nachweisen konnten [61].

Dies bedeutet zusammengefasst, dass die Therapie von Patienten mit Fazialisparese nicht nur Gesichtsfunktionen verbessern sollte, sondern die Patienten im Idealfall auch glücklicher aussehen lassen sollte. Hierfür müssen Konzepte und darauf ausgerichtete Evaluationskriterien entwickelt werden. Die gute Nachricht ist, dass kortikale und subkortikale Netzwerke des Gehirns sehr sensibel und schnell auf Veränderungen der Beweglichkeit reagieren und dadurch neuroplastische Prozesse zur Kompensation und Reorganisation initiiert werden [57].

## Ausblick

Wir haben hier eine sehr kurze Einführung in das Thema „Glück“ gegeben. Es sei erwähnt, dass die Thematik auch in der Ökonomie beträchtliche Aufmerksamkeit findet, besonders auch im Hinblick auf politische Entscheidungsfindung [62, 93]. Neben den hier zitierten psychologischen und neurowissenschaftlichen Werken ist die Frage nach dem Glück seit vielen Jahren Forschungsgegenstand in der positiven Psychologie [25].

Mit diesem Artikel wollen wir eine neue Sichtweise auf klassische Themen der HNO-Heilkunde anbieten und unsere Disziplin als wichtigen Partner in einem fachübergreifenden wissenschaftlichen Kontext vorschlagen.

## Fazit für die Praxis

- Wie wir hier kurz darzustellen versuchten, beschäftigt das Thema Glück die Denker und Wissenschaftler der geschichtlichen Epochen mindestens seit der Antike.
- Es ist erfreulich zu sehen, dass sich über die Jahrhunderte ein kohärentes Bild der unterschiedlichen Disziplinen und Ansätze ergibt: Zwischenmenschliche Beziehungen, kurzfristige und lockere Bekanntschaften („weak ties“) sowie natürlich tiefe, langfristige Freundschaften und familiäre Beziehungen sind ein zentraler Baustein für menschliches Glück und Gesundheit.
- Durch die Evolution ist das Gehirn perfekt dafür ausgerichtet, soziale Fähigkeiten und Fitness zu entwickeln.
- Andererseits arbeitet das auch gegen uns, weil wir auf Einsamkeit evolutionär nicht vorbereitet sind.
- Anhand von einigen Beispielen wurde erläutert, dass die HNO-Heilkunde innerhalb der medizinischen Fachbereiche eine zentrale Rolle einnimmt, um zu verstehen, wie grundlegende sensorische und motorische Prozesse (z. B. Hören, Riechen, Mimik) beitragen, soziale Fitness zu erreichen und bei Störungen wiederzuerlangen.
